# Assessing Vegetation Cover Dynamics Induced by Policy-Driven Ecological Restoration and Implication to Soil Erosion in Southern China

**DOI:** 10.1371/journal.pone.0131352

**Published:** 2015-06-26

**Authors:** Jien Zhang, Tianming Wang, Jianping Ge

**Affiliations:** 1 State Key Laboratory of Earth Surface Processes and Resource Ecology & College of Life Sciences, Beijing Normal University, Beijing, PR China; 2 Earth and Environmental Sciences Department, Lehigh University, Bethlehem, Pennsylvania, United States of America; Shandong University, CHINA

## Abstract

In the aftermath of the severe droughts and floods at the end of the 20th century, the Chinese government launched several ecological restoration projects, including the Natural Forest Protection Program in 1998 and the Grain-for-Green Program in 1999, to promote afforestation and reforestation to reduce surface runoff and consequent soil erosion nationwide. However, it is still unclear how vegetation has changed in southern China since the launch of these programs. In this study, we used the MODIS Enhanced Vegetation Index (EVI) to analyze the vegetation cover dynamics in southern China from 2000 to 2009 and evaluate the resulting effects of controlling soil erosion. Our observations indicate that 5.3% of the study area significantly increased and 0.98% significantly decreased in EVI value (*p* < 0.05). The spring EVI had largest increase in space. The conversions of croplands on steep slopes to forests resulting from national policies led to significant increases in EVI. The increase in EVI was not driven by annual average temperature and annual precipitation. By referencing ecological restoration statistical data and field observations, we showed that ecological restoration programs significantly improved vegetation cover in southern China. Increase in the area of farmland-converted forestlands has reduced soil erosion based upon monitoring sediment yields at hydrologic stations in the Yangtze River. This study displays the spatial patterns of trend in vegetation growth since the beginning of the 21st century in southern China and highlights the important role of China’s afforestation program.

## Introduction

Water loss and soil erosion have been deteriorating China’s environment and economy for a long time [1]. The economy in China, the largest developing country in the world, has experienced tremendous growth at the cost of environment degradation over the past thirty years [2]. Notable examples of the environmental degradation were the cut-off of the Yellow River for 226 days in 1997 and the massive flooding in the Yangtze River floodplain in 1998 [3]. In the wake of the huge damage caused by these natural calamities, the Chinese central government launched several ecological restoration projects since the late 1990s to promote afforestation and reforestation and reduce surface runoff and soil erosion nationwide, including the Natural Forest Protection Program (NFPP) Project launched in 1998, as well as the Grain-for-Green Program(GGP) in 1999 [4]. Many studies of these restoration projects and their resulting effects have been conducted at local scales, especially in the Loess Plateau of China [5–8]. Nevertheless, there is a lack of conclusive evidence that these managed practices positively influence vegetation cover and, consequentially, the control of soil erosion in southern China due to the complex interactions among climate, ecological, social and economic factors [9, 10].

Agriculture in China has a long history. Over the past 300 years, the area of agricultural lands in China increased from 6.8×10^5^ km^2^ in 1661, which was the early Qing Dynasty, to 9.6×10^5^ km^2^ in the 1990s; in comparison, forests shrunk from 2.5×10^6^ km^2^ in 1700 to 1.09×10^6^ km^2^ in 1949[11]. In the latter half of the 20th century, agricultural lands in China experienced significant fluctuations with apparent increases occurring from the 1950s to 1980s and slight decreases occurring after the 1980s [12]. Meanwhile, the forest coverage in China has increased since the 1950s because cropland has been converted back to forest or grassland in response to the government soil-conservation programs [13]. However, the destruction of natural vegetation in the upper and middle reaches of the Yangtze River has still been severe, leading to significant local water and soil erosion [14].

Surface runoff generation and soil erosion in southern China are highly influenced by land use and land cover (LULC) [15]. Integrative measures to reforest are key to prevent soil erosion on severely eroded bare land [16]. A 4-year (2000–2003) study showed that surface runoff and soil erosion decreased due to four forest restoration approaches in a hilly red soil region in Hengyang County, southern China [17]. In the same area, a further study showed that the four forest restoration approaches had significant effects on soil quality, which influenced the soil microbial biomass, as well as litter fall production rate [18]. In addition to LULC, slope plays an influential role on the soil erosion rates. A study conducted in Zhongjiang County of Sichuan showed that soil erosion was the highest on agricultural lands with slopes above 10° [19]. However, the overall conditions of vegetation dynamics and the resulting effects on water loss and soil erosion since the recent implementations of the nationwide ecological restoration projects have not been well studied in southern China, presenting a critical gap in data evaluation.

In this paper, we develop a top-down framework using the 250 m resolution MODIS EVI dataset through the incorporation of 30 m resolution Landsat TM/ETM+ images to study the vegetation dynamics and its driving forces in southern China from 2000 to 2009, during which the major restoration projects were implemented. We categorize our study into three components: 1) using remote sensing datasets to quantify the changes in spatiotemporal patterns of vegetation in southern China; 2) using meteorological, LULC, and restoration statistics data to detect the changes in the possible driving forces of vegetation cover dynamics; and 3) determining the potential ecological effects of the vegetation changes on soil erosion in the Yangtze River. Our findings are helpful in the decision making of vegetation reconstruction at regional scales in southern China.

## Data and Methods

### Study Area

**Fig 1:**
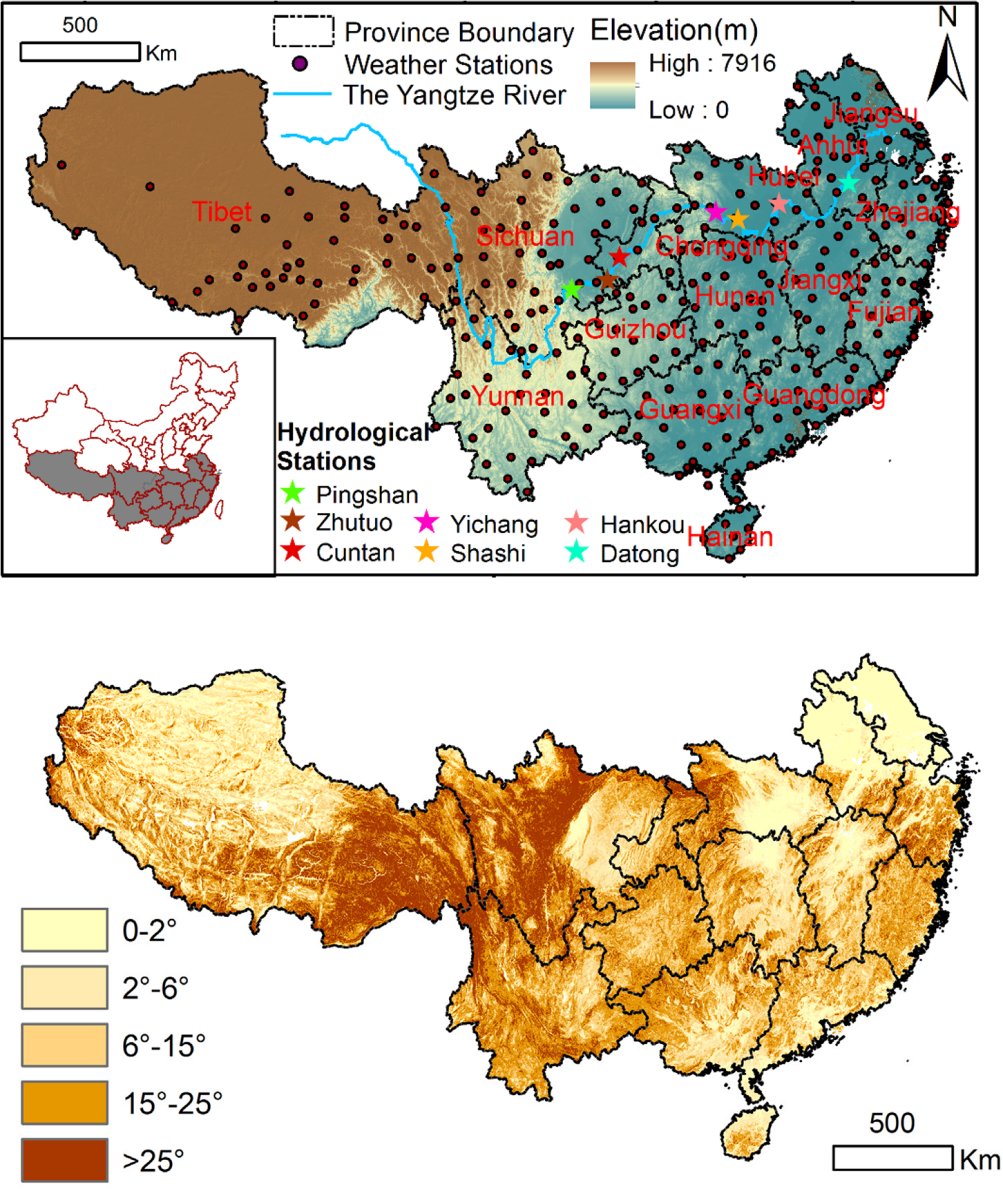
The elevation map, locations of meteorology and hydrological stations (above) and slope distribution in southern China (below).

The study was performed in southern China. It is located between 18°9′-36°29′N and 78°23′-122°57′E, covering a total area of 362.84×10^4^ km^2^. This region spans from the Tibetan Plateau, with an average elevation of 5000 m, to the East China Sea and crosses over 19 provinces in China (Fig 1). The population in this area accounts for approximately 58% of the total population in China (http://www.stats.gov.cn/tjsj/pcsj/). The typical climate of this region is dominated by a subtropical monsoon climate, with mean annual temperatures of 15–20˚C and an average annual rainfall of 1000 mm. The tropical climate in Hainan and the south boundary of Yunnan is characterized by mean annual temperatures of 21–25˚C and an annual rainfall up to 1200 mm. The climate on the Tibetan Plateau is a typical highland climate with a mean annual temperature of 3˚C and annual rainfall of 300 mm. The geomorphology in the study area varies. The Yangtze River is the most important water resource for southern China and crosses the whole region with a number of tributaries. The upper reaches of the Yangtze River are mainly mountainous. In the southwest portion of the study area, karst landforms are dominant, such as in Guangxi and Guizhou. The middle-lower reaches of Yangtze River are a major floodplain and prime land for agricultural activities. The rest of the study area consists of mountainous land and plains.

Forests and croplands are the major vegetation types in southern China. The deciduous broad-leaf forest is distributed in the northern part of the study area with yellow-cinnamon soils, including Hubei, Anhui, and the southern portion of Jiangsu. The ever-green broad-leaf forest in southern China has two sub-areas: the eastern sub-area with ever-green forest with red earth soils dominates the coastal areas, as well as the hills in the lower reaches of the Yangtze River, and the western sub-area with ever-green and coniferous forest with yellow earth soils includes the Sichuan Basin and Yungui Plateau. The tropical forest in the southernmost part of the study area, including Guangdong, Guangxi, Yunnan, and Hainan, has humid-thermo ferralitic soils. The ecosystems on the Tibetan Plateau from west to east are Alpine steppe, Alpine meadow, and shrubland with frigid desert soils. In addition, the fragmented agricultural lands permeate the southwestern karst regions, southern forest lands and eastern coastal hills. GGP covers 11 provinces, including Anhui, Jiangxi, Hubei, Hunan, Guangxi, Hainan, Chongqing, Sichuan, Guizhou, Yunnan and Tibet. Crop fields and barren lands on the slopes are the particular foucs of the restoration projects. Tree species selected for reforestation planted include *Pinus armandii*, *Pinus massoniara*, *Cryptomeria fortune*, *Cunninghamia laceolata*, *Cupressus* spp., *Eucalyptus* spp. and *Populus deltoids*.

### MODIS EVI

The 2,260 composite 16-day MODIS EVI images used in this study were downloaded from the NASA Earth Observing System Data Gateway (https://lpdaac.usgs.gov/) for the period of March 2000 to December 2009. The MODIS EVI tiles were processed to 250×250-m resolution by running the MODIS Re-projection Tool (MRT) from the same website. After that, 10 composite MODIS EVI images with the same record date, which covers the entire study area, were merged into one image. For each year, there are 22 or 23 merged images. We stacked these merged images into one image, which contains 22/23 ordered EVI values, for each year. Then these images were first subset to the study boundary using ERDAS Imagine 9.2. The annual integrated EVI was selected as a proxy for vegetation production. Its values for each grid were computed as averages of the growing season of each year (April to November). Finally, the 10 composite growing-season average EVI images were stacked, filtered and noise-reduced for trend analysis and land classification [20, 21]. The annual integrated EVI time series were analyzed using ordinary least squares (OLS) and standard t-test at a confidence level of 95% to detect significantly positive and negative trends [22–24].

### Meteorology Data

The meteorological data used in this study includes daily temperature and precipitation data, which were collected from 320 weather stations provided by the China Meteorological Data Sharing Service System (CMDSS) (http://cdc.cma.gov.cn) from 2000 to 2009. Monthly average and annual average temperatures, as well as monthly and annual precipitation, were calculated. The Mann-Kendall (MK) non-parametric method was used to test the significance of annual average temperature and annual precipitation trends [25, 26]. To determine the relationship between climate and EVI, we calculated the Pearson Correlation Coefficient between the 10-year EVI time series and characterized the annual average temperature and annual total precipitation time series. We first found the weather stations with significant changes in annual mean temperature and annual total precipitation. We then created 1 km×1 km buffers centered on those stations. After that, we extracted the EVI time series within the buffers. Finally, these extracted EVI time series and the meteorological time series of the corresponding weather stations were applied for the Pearson Correlation calculation.

### Land-use Classification

The land classification map of 2009 was created using the unsupervised classification method applied to the growing season EVI stack. Thirty LULC types were created first and then merged into 6 types (water body, forest, shrubland, cropland, crop-natural vegetation mosaic and grassland) referencing topographic maps. To verify the classification accuracy, Kappa Coefficients were calculated referencing Google Earth Maps [27, 28]. In this process, 188 points were randomly created and overlaid on the land classification map of 2009 using ArcGIS 9.3. The Kappa Coefficient of 2009 land classification was 0.86. For the LULC classifications of 2000, we still adopted the unsupervised classification. The thirty LULC types that were the same as for 2009 were narrowed down to 6 types. After that, the National Land Cover Dataset of 2000 (NLCD-2000) provided by the Chinese Academy of Sciences was also used for verification due to its temporal comparability.

The SRTM 30 m DEM datasets were adopted for slope analysis on the grids with significantly changed EVI. We first categorized the slope into five classes (0–2°, 2°-6°, 6°-15°, 15°-25°, and >25°) and then overlapped the slopes with the significantly increased EVI. We then merged the five slope classes to 0–15° and >15° because land on a 0–15° slope is appropriate for agriculture development and land on >15° is soil-erosion significant. We then extracted the significantly increased EVI and land cover maps of 2000 and 2009 by the two slope classes to observe the vegetation changes in terms of slopes.

### Vegetation Change Validation

A validation was carried out by comparing EVI trend maps with ground-based points from field trips. We took photos to document the ground true sites and landscapes by using GPS cameras. Geo-referenced field photos were collected during our field trips in 2010. In addition, field photos and associated land cover information, which covered the most EVI increased area, were provided by local forest departments in the past few years (S1 Fig).

To further validate the detailed land cover changes, we selected a typical area with the most significant EVI increases and mapped the LULC using one Landsat-7 TM/ETM+ image for 2002 and 2010. A raster format of land classification was obtained using a supervised classification method. LULC types include water body, agricultural land, crop-natural vegetation mosaic, and forest according to the IGBP LULC classification system. In addition, the Kappa Coefficient was calculated for the 2010 land cover map by creating 250 random sampling points. The Kappa Coefficient was 0.77. The 2002 LULC map was created using the same approach as for 2010.

### Hydrological Data

We adopted the hydrological data of seven monitoring sites along the main reaches of the Yangtze River (Fig 1) to study the effects of vegetation restoration on water and soil loss on a large scale. The hydrological data includes runoff volume and sediment yield. The temporal scale of most hydrological data spans from 2000 to 2011 [29]. These stations monitored the whole restoration area.

### Statistical Data

Statistical data on ecological restoration was collected from local forestry departments and the State Forestry Administration of China. The majority of the statistical data assessed was the GGP from 1999 to 2008 and includes the total area of recovered cropland, converted cropland, fenced-off lands, and afforested lands. The statistical data for the NFPP only includes the total area of protected land. There were areas of overlap between these two forestry programs (GGP and NFPP), and the respective scope for each was difficult to distinguish; therefore, we integrated these two projects to consider just the general ecological restoration. Specifically, we mainly applied the GGP statistical data due to its comprehensiveness but referenced the NFPP statistical data to ensure proper correspondence.

## Results

### Spatiotemporal Trends of Vegetation Changes

**Fig 2:**
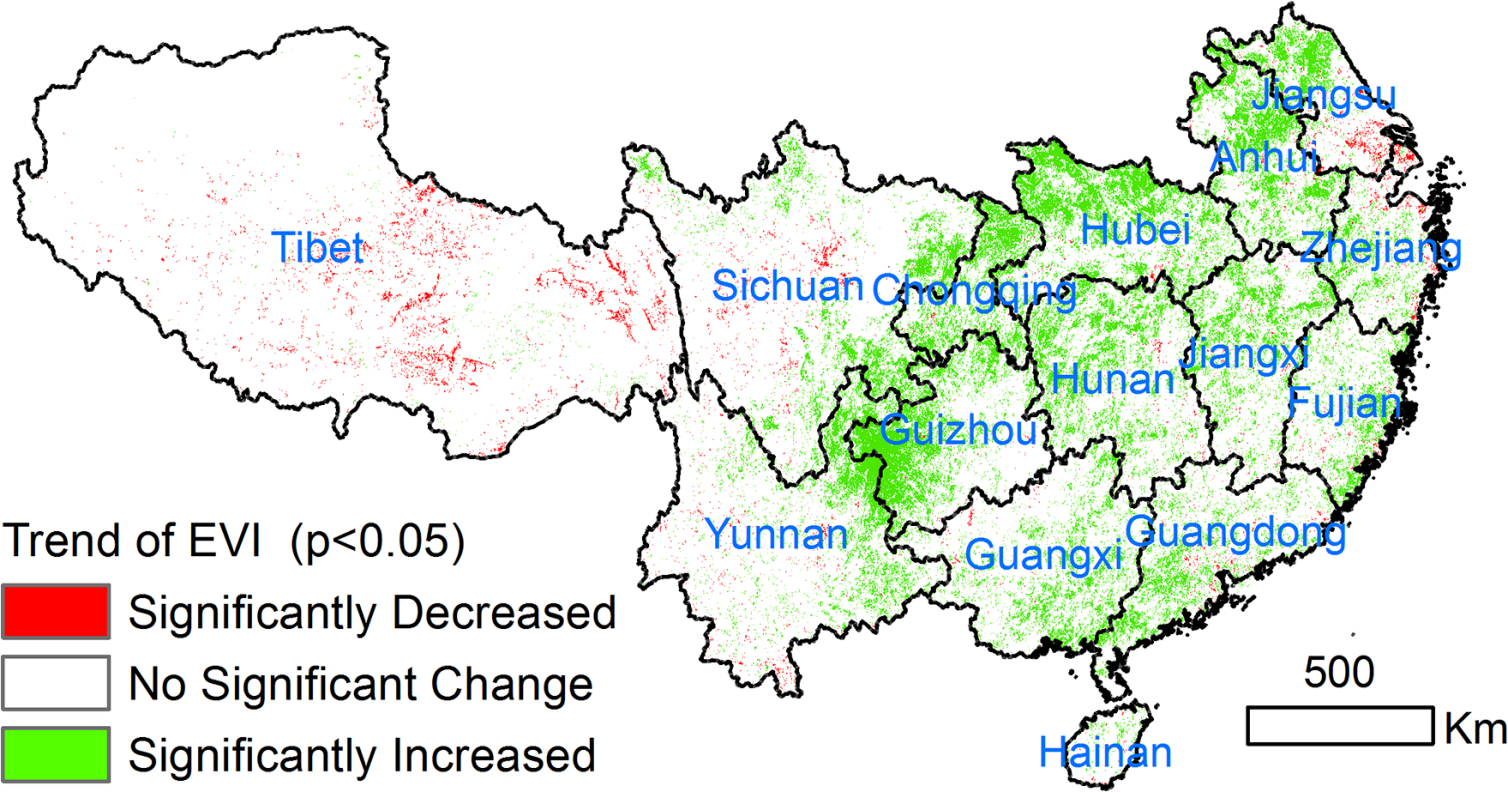
The significant changes in EVI for the period of 2000–2009 (*p*<0.05).

Significant increases in EVI account for 5.3% of the entire study area from 2000 to 2009 (*p <* 0.05), mainly found in the eastern part of southern China. Significant decreases in EVI occurred in 0.98% of the study area (*p* < 0.05), distributed throughout the Tibetan Plateau, Sichuan Basin, and some costal places (Fig 2).

**Fig 3:**
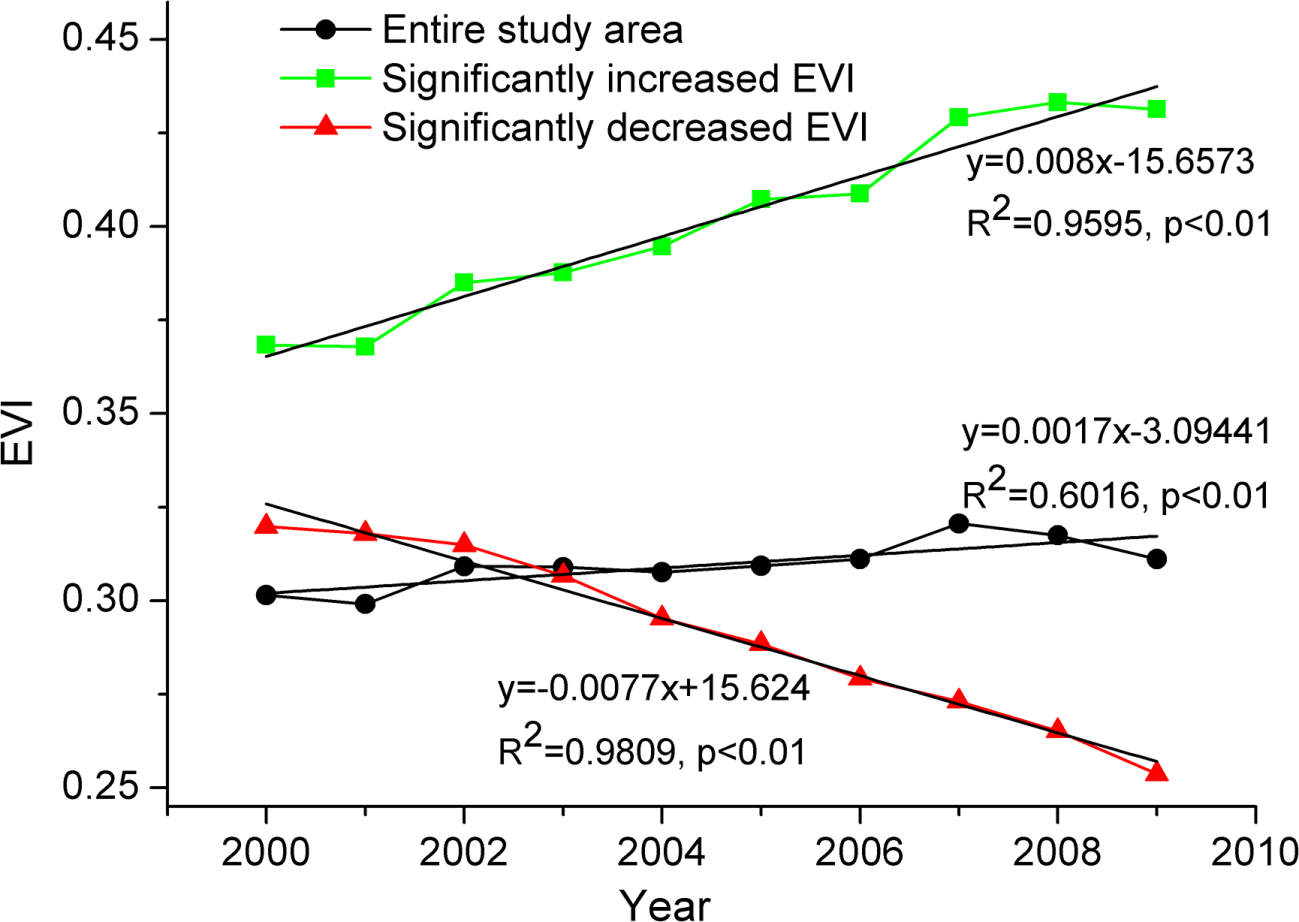
Time series of averaged EVI in southern China. Black is for the entire study area, green is for the regions with significantly increased EVI, and red is for regions with significantly decreased EVI.

Fig 3 shows changes in the time series of EVI over the ten years. For the entire area of southern China, the temporal trend of EVI significantly increased (R^2^ = 0.60, *p* < 0.01) with a maximum value of 0.33 in 2007 and a minimum value of 0.30 in 2001. In the area of significantly increased EVI, the rate of EVI increase was 0.008 (R^2^ = 0.96, *p* < 0.01). In the area of significantly decreased EVI, the decrease rate was -0.0077, and the decrease in EVI was persistent and significant (R^2^ = 0.98, *p* < 0.01).

### Seasonal Vegetation Anomalies

To better understand the seasonal changes of EVI, we separated the growing season into three seasons, spring (April and May), summer (June, July, and August), and autumn (September and October). The same processing approach as for the annual EVI changes was applied for each season’s EVI. The seasonal changes in EVI are shown in Table 1. Seasonal EVI show a high degree of spatial heterogeneity and all three seasons contributed to the increased in EVI. In the regions with significantly raised EVI, the largest increases occurred in the spring accounting for 39.69% of the total EVI increase areas, followed by the summer (20.59%) and autumn (17.24%) (Table 1 and S2 Fig). The rest of the significant increases in EVI were dominated by a combination of spring, summer, and autumn, which represented less than ten percent of the total EVI increases. In the area with significantly decreased EVI, summer and autumn dominated the decreases in EVI, accounting for 46.92% and 27.69%, respectively; meanwhile, spring accounted for 13.56%. The combined EVI decreases over spring, summer and autumn were less than 5%.

**Table 1:** The area and percent of EVI increase or decrease at the 5% significance levels on total significant EVI increase areas for different season and combination of seasons.

	Significantly increased EVI		Significantly decreased EVI	
	Area (10^4^ km^2^)	%	Area (10^4^ km^2^)	%
**Spring**	32.88	39.69	2.86	13.56
**Summer**	17.06	20.59	9.89	46.92
**Autumn**	14.28	17.24	5.83	27.67
**Spring + Summer**	6.51	7.86	0.41	1.95
**Spring + Autumn**	5.79	6.99	0.38	1.82
**Summer + Autumn**	3.43	4.14	1.05	4.98
**All three seasons**	2.88	3.48	0.66	3.11

### Driving Forces of Vegetation Changes

#### Climate Changes

**Fig 4:**
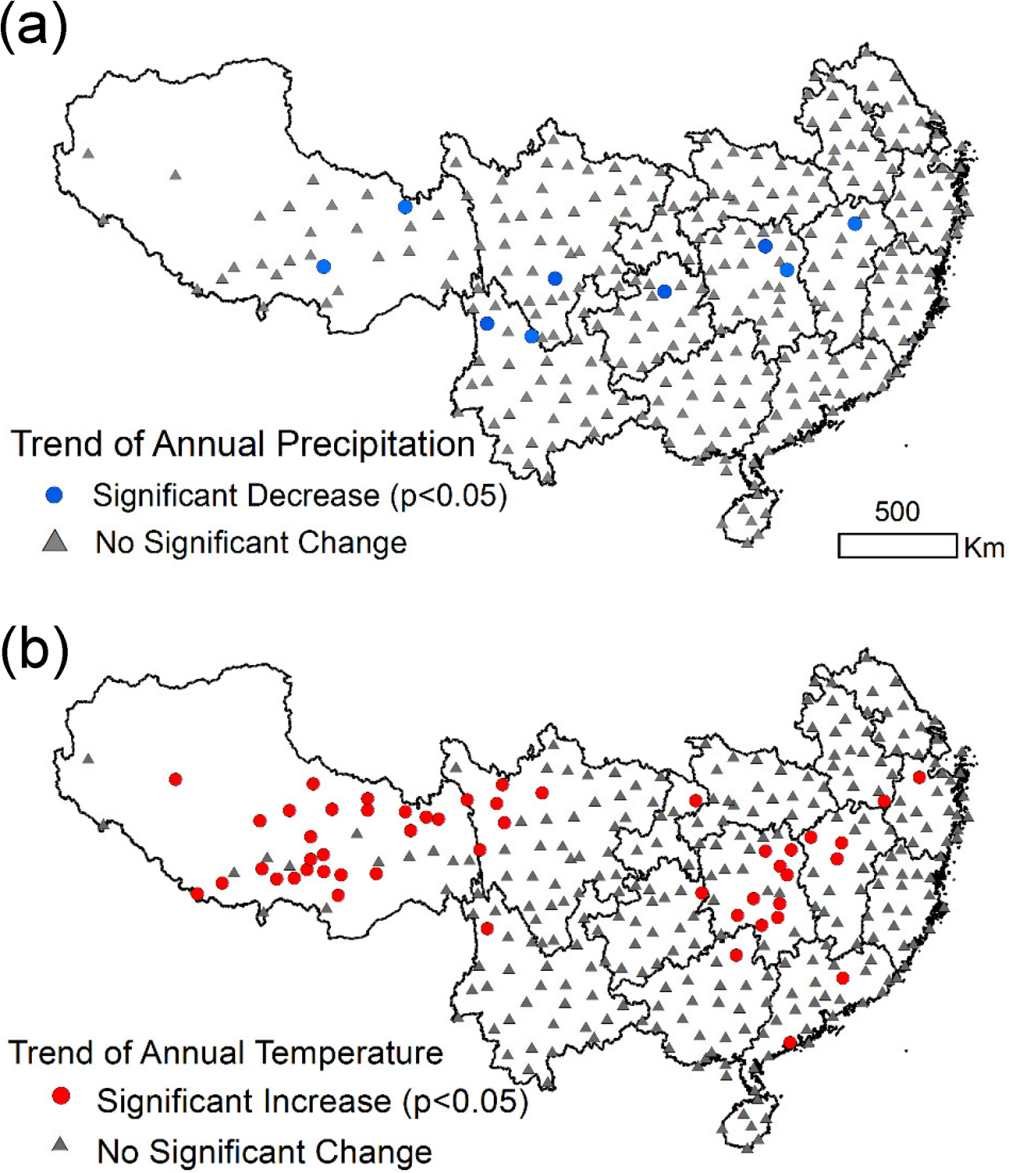
Trends of annual average temperature change (a) and annual precipitation change (b) for the period of 2000–2009 (*p*<0.05).

The Mann-Kendal test for the weather stations from 2000–2009 shows that 53 stations had significant increases in temperature (Fig 4A). Most stations with a significant increase in annual temperature were clustered in the Tibetan Plateau. The remaining weather stations with significant annual temperature changes do not show any spatial correspondence to the areas of significant EVI changes. We extracted the annual EVI from a 1-km buffer area around the sites with significant increases in temperature and conducted a correlation analysis between the EVI and annual average temperature. The results show that only 3 sites have significant correlations (*p* < 0.05).

The Mann-Kendal test indicates 9 weather stations had significant decreases in annual precipitation (Fig 4B). These nine stations did not correspond to the EVI spatial pattern. In addition, none of the 9 stations had significant correlations between the EVI and annual precipitation within the 1-km buffer area. All other stations did not have significant changes in annual precipitation from 2000 to 2009. Based on these analyses, it is clear that the air temperature and precipitation are not strong contributing factors to the significant increase in EVI in restoration areas.

#### Land Cover Changes

**Fig 5:**
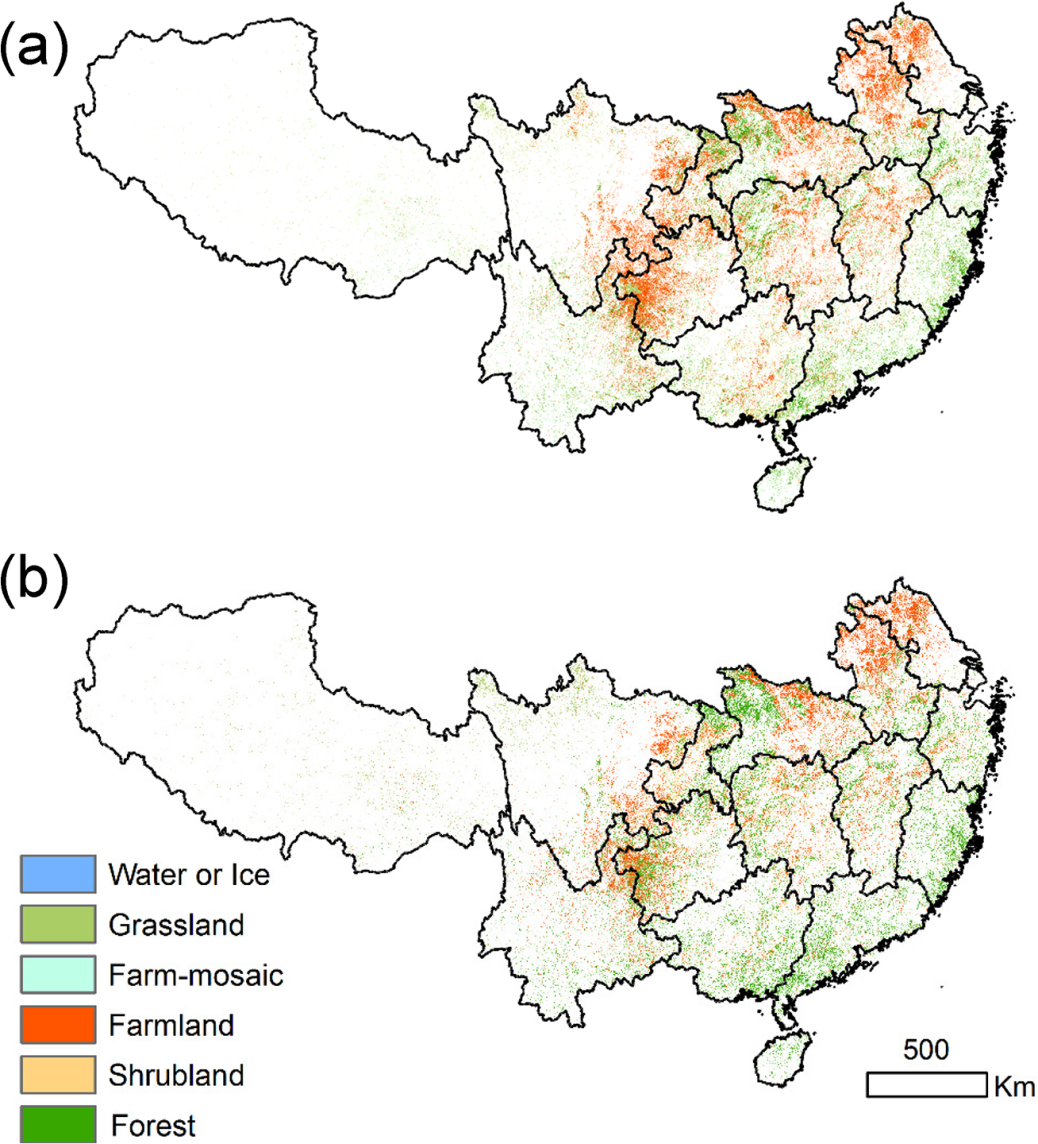
The land use and land cover in the area with significantly increased EVI in 2000 (a) and 2009 (b).

We extracted the land cover of 2000 and 2009 by overlapping the maps of significantly changed EVI (Fig 5). In both 2000 and 2009, cropland dominated Anhui, Jiangsu, northern Hubei, central Hunan and Jiangxi, which are the main grain-producing areas of China. Trends in agricultural practices, such as increased use of high-yield crops and chemical fertilizers use probably made a contribution to the greening trend in these regions. Furthermore, the area of forest in Zhejiang, Fujian, and Guangdong increased to some degree. The area of cropland decreased in southwest Guizhou, northeast Yunnan and southeast of Sichuan. These areas are the typical eco-fragile region in China with karst topography and thin soil layer. The areas of cropland that were converted to grassland, shrubland, and forest were 3.16×10^4^ km^2^, 4.16×10^4^ km^2^, and 5.29×10^4^ km^2^, respectively (Table 2). The net decrease of cropland was 9.58×10^4^ km^2^. The net increases of forest, shrubland, and grassland were 3.98×10^4^ km^2^, 3.44×10^4^ km^2^, and 1.53×10^4^ km^2^.

**Table 2:** Change in land use area (10^4^ km^2^) in the area with significantly increased EVI and as a percentage of total area changed (numbers in parentheses), between 2000 and 2009.

2000 to 2009	Grassland	Crop-Natural Vegetation	Cropland	Shrubland	Forest	Total
**Grassland**	3.12 (59%)	0.62 (12%)	1.39 (27%)	0.01 (0%)	0.11 (2%)	5.25
**Crop-Natural Vegetation**	0.13 (12%)	0.34 (30%)	0.50 (44%)	0.01 (1%)	0.14 (13%)	1.12
**Cropland**	3.16 (12%)	0.71 (3%)	13.53 (50%)	4.16 (15%)	5.29 (20%)	26.85
**Shrubland**	0.04 (1%)	0.00 (0%)	0.03 (1%)	0.37 (10%)	3.31 (88%)	3.75
**Forest**	0.33 (2%)	0.08 (0%)	1.82 (11%)	2.64 (15%)	12.2 (71%)	17.07
**Total**	6.78	1.75	17.27	7.19	21.05	54.04

**Fig 6:**
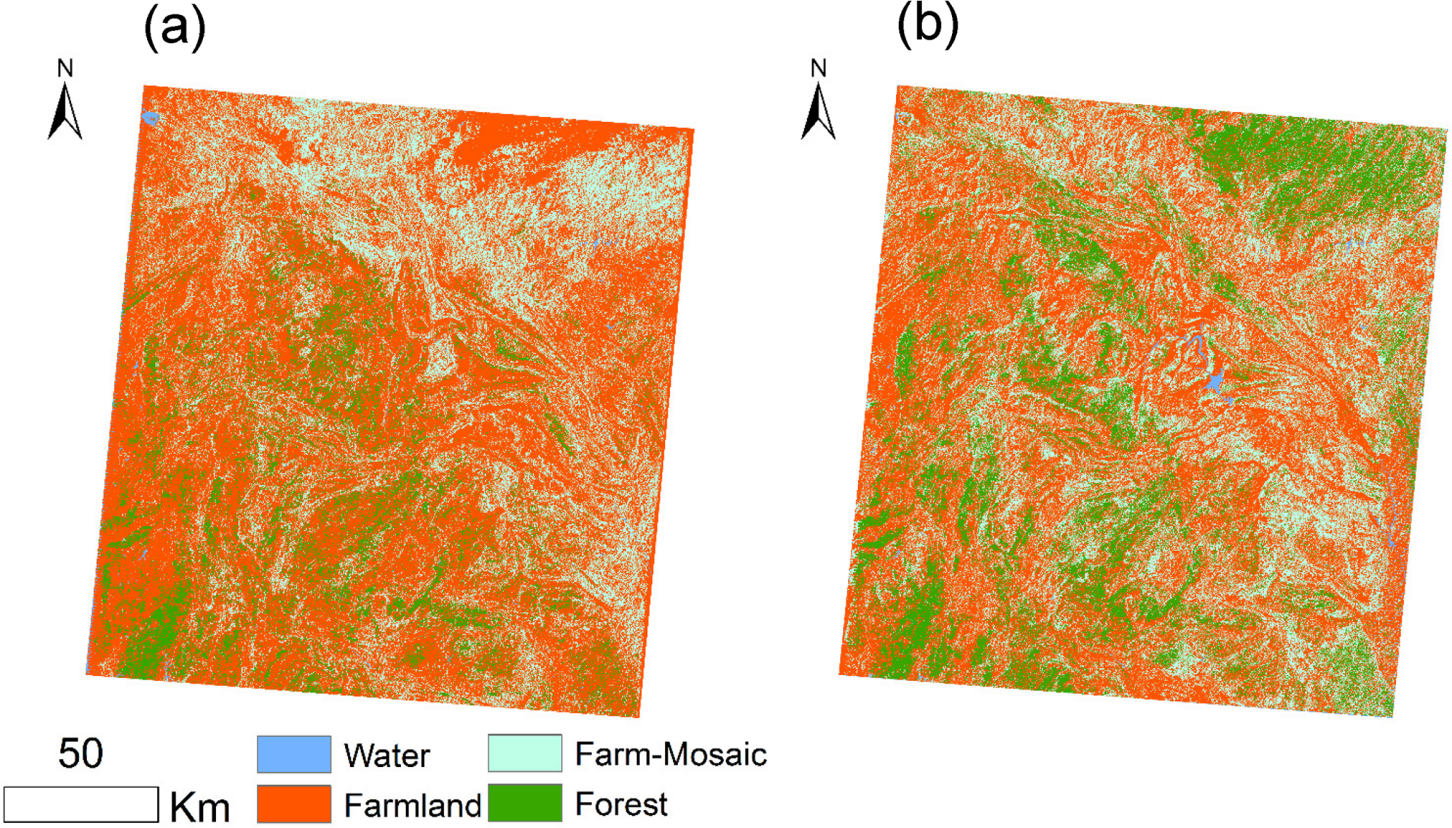
The land use change of the typical area with the significant EVI increase in 2002 (a) and 2010 (b). It locates the boundary area among Sichuan, Yunnan and Guizhou.

We selected the transitional area among Sichuan, Yunnan, and Guizhou as the typical zone of vegetation changes. Although cropland was still dominant in 2009, it was highly fragmented, indicating LULC was transforming to forest and other natural vegetation types (Fig 6). The LULC obtained from the Landsat TM/ETM+ images of this region for 2002–2010 show that the forest and natural vegetation increased by 2.3×10^3^ km^2^ and 1.56×10^3^ km^2^, while cropland decreased by 3.9×10^3^ km^2^.

#### Slope Distribution of Changed Land Cover Types

**Fig 7:**
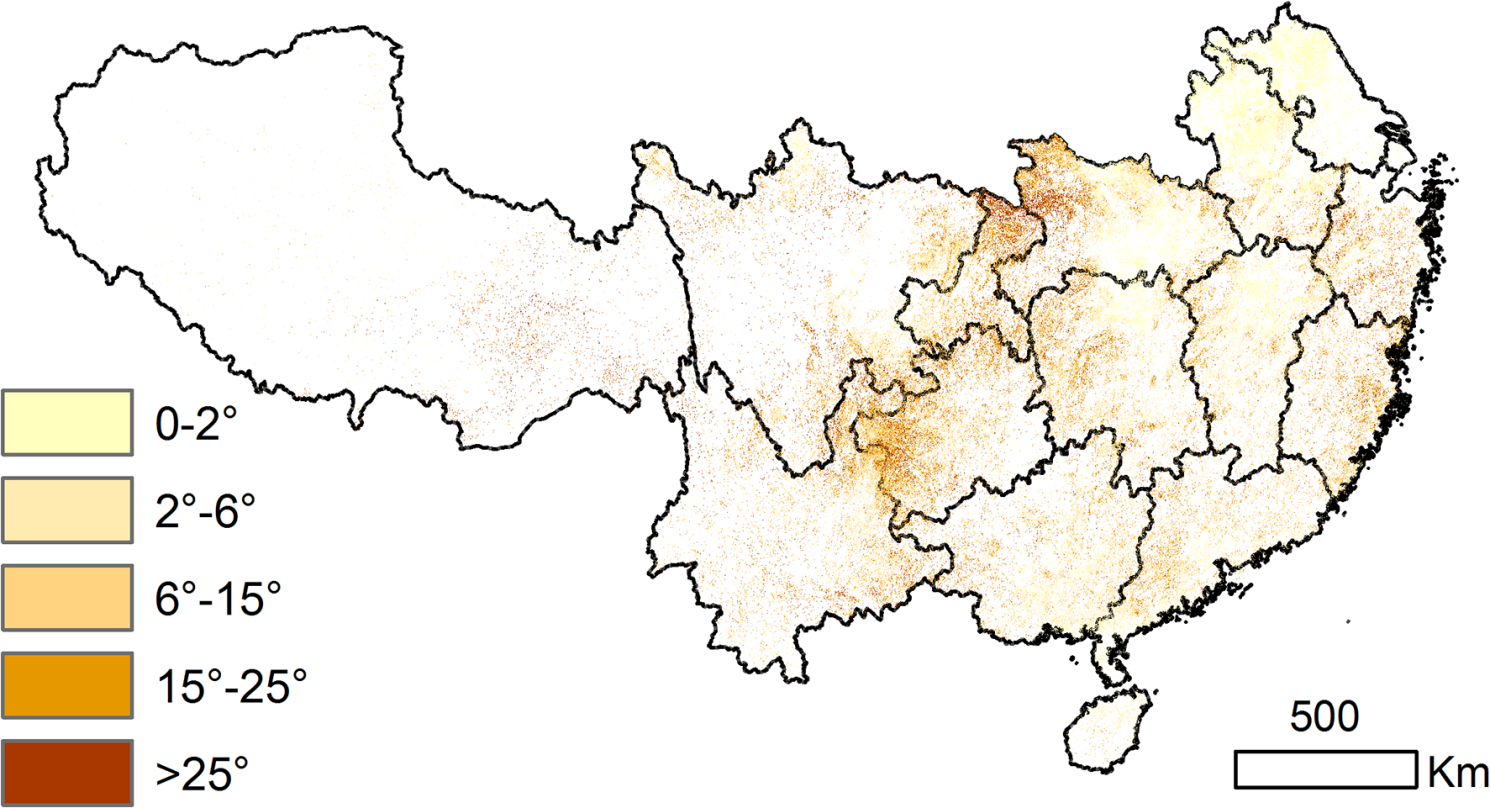
The slope distributions in the area with significantly increased EVI 2000–2009.

The slope distribution for southern China is shown in Fig 1. Slopes of 0–15° account for 68.6% of the area with significantly increased EVI, and 31.4% of the area occurs on slopes above 15°. Significantly increased EVI on slopes of 0–15° occurs in the northeastern (i.e., Anhui, Jiangsu, and north of Hubei), central-eastern (i.e., north of Jiangxi and Hunan) and southern (i.e., south of Guangxi and Guangdong) parts of the study area; significantly increased EVI on slopes above 15° occurs in the central (i.e., northwest of Hubei, north of Chongqing, and central-west of Hunan), southwestern (i.e., southwest of Guizhou and northeast of Yunnan), and eastern (i.e., Zhejiang, southeast of Fujian, and southwest of Guangdong) parts of the study area (Fig 7).

**Fig 8:**
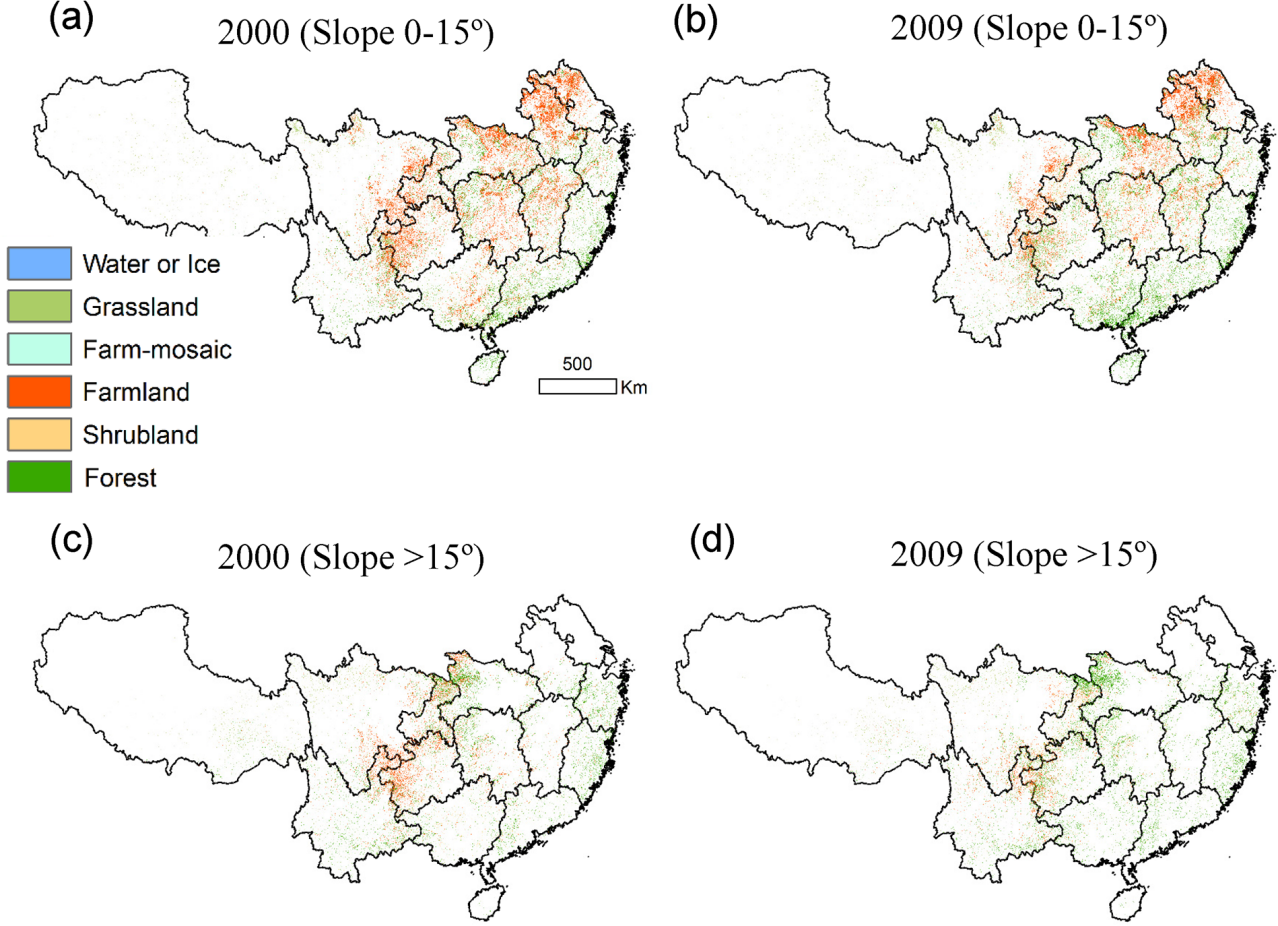
The land use for the area with significantly increased EVI on slopes of 0–15° in 2000 (a) and 2009 (b) and on slopes >15° in 2000 (c) and 2009 (d).

According to the two slope categories, the LULC types of the area with significantly increased EVI for both 2000 and 2009 are shown in Fig 8 and S3 Fig. Agricultural land was the dominant type on slopes of 0–15° in 2000. On slopes of 0–15° cropland decreased by 6.5×10^4^ km^2^ and forest, shrubland, and grassland increased by 2.5×10^4^ km^2^, 1.6×10^4^ km^2^, and 1.0×10^4^ km^2^, respectively, from 2000 to 2009 (S3 Fig). The region with the most apparent change is north of Chongqing and the transitional zone of Sichuan, Yunnan and Guizhou, where cropland was significant in 2000 but decreased in 2009. On slopes above 15°, cropland decreased by 3.0×10^4^ km^2^, and forest, shrubland, and grassland increased by 1.0×10^4^ km^2^, 1.0×10^4^ km^2^, and 0.5×10^4^ km^2^, respectively. However, in the northern part of Chongqing and the transitional zone of Sichuan, Yunnan and Guizhou, the forest increased and cropland decreased significantly. The local forestry administrations provided the information of land cover of selected sites where we conducted field investigation and took 520 field photos. The data indicated that the vegetation recovered significantly after the restoration policies were carried out in these regions (S1 Fig).

### Effects on water and soil loss

**Fig 9:**
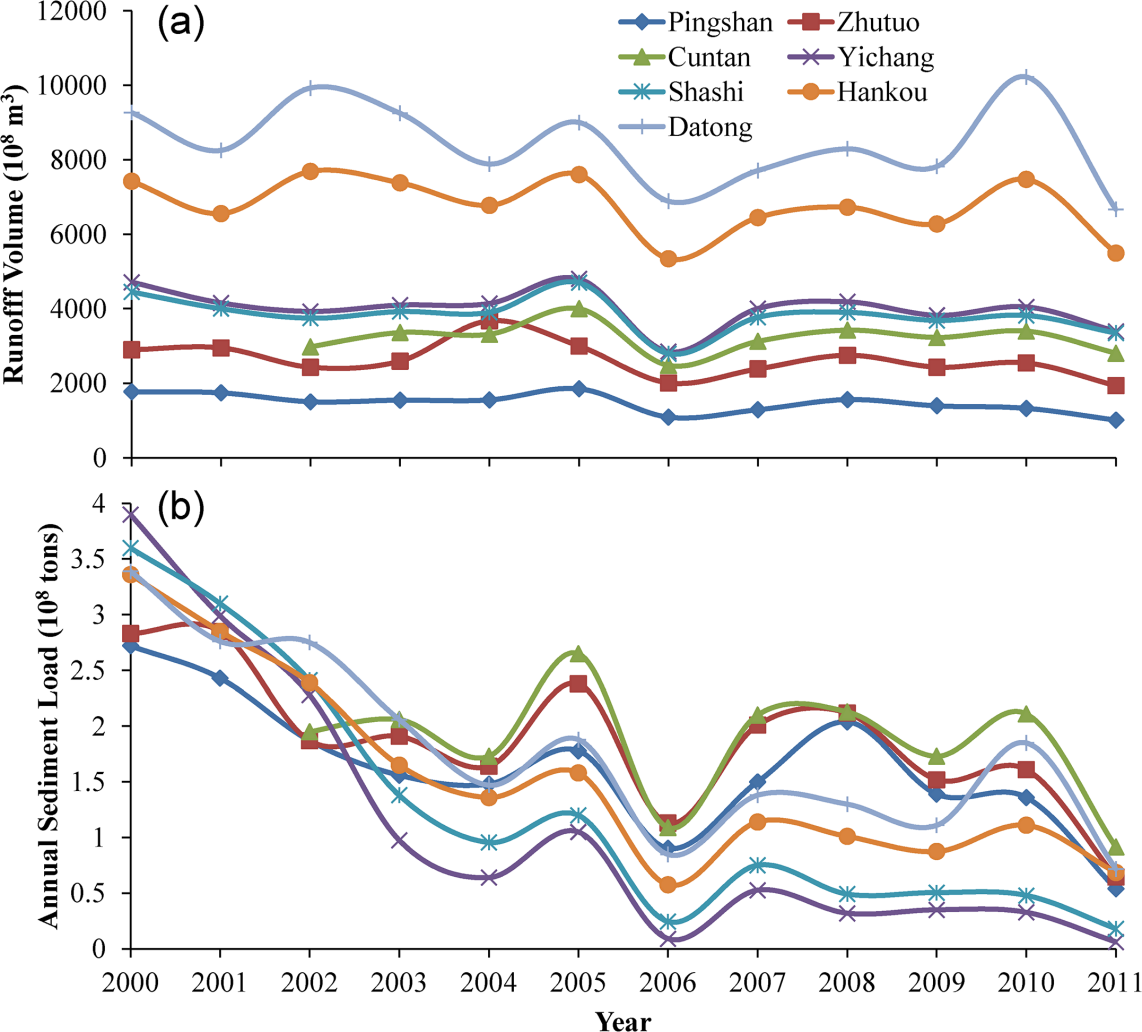
Time series of runoff volume (a) and sediment yield (b) measured at seven hydrological stations along the main stream of Yangtze River.

We evaluated the runoff volume and sediment yield in the gauging stations to determine if an obvious decrease occurred in response to the recent changes in vegetation. The average runoff volume of the seven stations shows a decreasing but not significant trend (R^2^ = 0.32, *p* > 0.50) (Fig 9A). Records of runoff volume from all stations in 2006 were low due to the shortage of precipitation that year. The average sediment yield at the seven hydrologic monitoring stations show a significant decrease (R^2^ = 0.72, *p* < 0.01) while no precipitation trends were observed for this period (Fig 9B). From 2000 to 2004, the decreasing trend of sediment yield was significant and persistent. However, the sediment yield increased sharply at all stations in 2005, which may be the result of high precipitation from the strong El Niño year. The sediment yield of all stations in 2006 was relatively low due to the correspondingly less precipitation. From 2007 to 2011, all seven stations showed a decreasing trend in sediment yield, albeit less significant than that from 2000 to 2004.

## Discussion

### Effects of eco-restoration projects on vegetation

Since the 1970s, the Chinese government launched a series policies and projects towards ecological restoration. The largest land retirement program and the most ambitious among these projects were the GGP, begun in 1999 to convert cropland on steep slopes to natural vegetation and afforestation of barren land by providing farmers with grain and cash subsidies [4]. The project has produced substantial environmental benefits, such as tree cover increase[30–32], erosion reduction[33] and carbon sequestration [34]. The statistical data showed significant conversions of croplands to forests under GCP in southern China (Table 3). The area of crop fields on the slopes (>5°) in southern China totals 11.27×10^4^ km^2^, accounting for 63.11% of total slope agricultural fields in the whole country. From 2000 to 2009, an area of 10.12×10^4^ km^2^ was reforested under the GGP. The area of 3.67×10^4^ km^2^ slope farmlands had been transformed to forest, being about 33% of total slope lands in southern China.

**Table 3:** The area of slope cropland and the statistical area of Grain-for-Green Program for the major provinces in southern China.

Provinces	Slope cropland Area (10^4^ km^2^)	Percent of slope cropland in China (%)	Area of Reforestation (10^4^ km^2^)			
			Total area	Reforestation of cropland	Afforestation of barren land	Forest reservation
**Anhui**	0.24	1.33	0.60	0.22	0.32	0.07
**Jiangxi**	0.45	2.54	0.66	0.20	0.39	0.07
**Hubei**	0.93	5.23	0.99	0.33	0.56	0.09
**Hunan**	0.42	2.35	1.31	0.50	0.71	0.10
**Guangxi**	0.45	2.54	0.90	0.23	0.58	0.09
**Hainan**	0.0083	0.05	0.17	0.04	0.12	0.01
**Chongqing**	1.19	6.65	1.17	0.44	0.65	0.08
**Sichuan**	2.14	11.98	1.89	0.89	0.89	0.10
**Guizhou**	2.32	12.97	1.25	0.44	0.70	0.11
**Yunnan**	3.07	17.18	1.09	0.36	0.64	0.10
**Tibet**	0.052	0.29	0.08	0.02	0.06	0.00
**Total**	11.27	63.11	10.12	3.67	5.62	0.83

Large-area increase in EVI occurred in the middle part of the study area (Chongqing, Sichuan, Guizhou and Yunnan) where large afforested area were observed (Table 3). The four provinces with 48.78×10^4^ km^2^ slope farmland account for 77% of total slope lands in southern China (Table 3). These regions have the largest continuous karst topography in China [35]. Due to the thin soil depth, which has limited capacity for agricultural activities, and huge population pressure, they suffers serious rock desertification and soil erosion [36]. In addition, these regions has the largest amount of steeply sloped land (>15°) compared with the other provinces in southern China. According to the restoration statistics, the GGP area in these regions was 5.4×10^4^ km^2^, which covers approximately 62% of the sloped cropland distribution (Table 3). Due to the implementation of an ecological afforestation project, some cropland, grassland and shrubland has been transformed to forest, and this conversion mainly contributed to forest restoration as approximated by EVI from 2000 to 2009. As a result, the role of the GGP is much higher than in the other provinces, and the vegetation increases are the most significant in these regions (Fig 2), coinciding with the higher level of GGP activity. LULC transitioned from agricultural land to natural vegetation, and the slope distribution of the land cover changes is mainly >15° (Fig 8C and 8D). We collected field photos and interviewed the local farmers and forestry departments in 2010 to verify results shown by remote sensing. From our field validations, it was clear that natural vegetation had recovered since the beginning of GGP in 2000 (S1 Fig). In addition, fencing for reforestation was adopted and felling was forbidden. Local government has been recovering the barren mountain through aerial seeding, which significantly helps vegetation recovery. Large numbers of working age people have moved out of the region, which has promoted forest increase by reducing negative disturbance of forest areas and promoting abandonment of sloping cultivated land[37]. In summary, both satellite-derived EVI and forestry statistical data showed that vegetation in southern China substantially increased since the launch of national ecological restoration policy in 1999. Previous study has shown that locations and extents of afforestation detected by remote sensing are highly consistent with forestry statistical areas which indicated the high performance of GGP [32]. The trend analysis of NDVI data found that ecological afforestation projects resulted in general greening trends of the forests in Guizhou and Hunan province from 2000 to 2010[32, 37]. Despite mild climate change in southern China, the increase in vegetation was not correlated with temperature or precipitation. Given forest have higher GPP than farmlands[31], the ongoing conversion of farmlands to forest under GGP had increased significantly to the carbon sequestration [34, 38, 39] in southern China. This indicates that the effort in converting farmlands on steep slopes to forests has been generally effective.

### Effects on water and soil loss

Our result showed that significant vegetation improvement had occurred in southern China, particularly in croplands with the steep slopes, which could make an important contribution to decrease in soil erosion. Previous studies showed that the GGP had led to significant decrease in runoff and soil erosion because of an increase in the area of farmland-converted forestlands in the Yangtze River [33, 40], confirming our findings. In addition, many studies have evaluated the effects of the afforestation and reforestation on soil erosion in southern China. Hill and Peart (1998) systematically reviewed the relationship between land use, runoff, and erosion for southern China based upon widespread plot studies [15]. They found that forests and woodlands have the lowest average rates of erosion, approximately 5 t km^-2^ year^-1^, and cultivated slope-land and bare soil experience the greatest erosion with mean erosion rates of 6,240 and 15,300 t km^-2^ year^-1^. For cultivated lands, the most serious soil erosion is observed on slopes of 10–25° in Zhongjiang (105°00′E, 31°01′N), a typical agricultural county of Sichuan Province [19].To decrease soil erosion through forest restoration has been nationwide efforts in China since the 1950s. For example, soil erosion was reduced to 200–4,300 t km^-2^ year^-1^ in 1990 from 5,300–25,600 km^-2^ year^-1^ in 1988 due to the reforestation in Yujiang County (116°55′E, 28°15′N) of Jiangxi Province [16]. In a hilly red soil region in southern China, plantations of slash pine (*Pinus elliottii*), Chinese fir (*Cunninghamia lanceolata*), tea-oil camellia (*Camellia oleifera*), and natural secondary forest decreased surface runoff by 63.0–88.1% and soil erosion by 75.5–97.1% after long-term regenerations of forest cover; the runoff and soil erosion in tea-camellia plantation and natural secondary forest plots were significantly lower than that of the other treatments [17]. To mitigate soil erosion by converting croplands on steep slopes is the most important focus of the ecological restoration projects. These results suggest that the forest cover improvement induced by the policy in southern China during the past 10 years has started to have measurable positive impacts on soil erosion of steeply sloped lands. To have better understanding of the relationship between vegetation change and sediment load, it is essential to distinguish the complex interactions among climate, ecological factor and human activities using erosion model. However, this study was not capable of distinguishing these factors. Our future studies will definitely pay more attention to the spatial context of these processes.

## Conclusions

The combination of MODIS EVI datasets, Landsat TM/ETM+ images, restoration statistics data and field validation provides a comprehensive framework to analyze the vegetation dynamics. We found that the ecological rehabilitation programs have positively contributed to increasing the vegetation cover in southern China for the period 2000 to 2009. Our results revealed that significant increases in EVI represented 5.3% of the study area. The spring EVI had largest increase in space. The conversions of croplands on steep slopes to forests resulting from national policies led to significant increases in EVI. The increase in EVI was not driven by annual average temperature and annual precipitation. The effects of the vegetation increases on the control of soil loss were positive based upon the monitoring of sediment yields in the main stream of the Yangtze River. These results provide a better understanding of and new insight into vegetation restoration in degraded forest ecosystems at regional to sub-continental scales.

## Supporting Information

S1 Fig Photos illustrating the afforestation effects in Hunan province, the major Grain-for-Green Program region of southern China. a, Less productive agricultural fields on the slopes before 2001 with severe soil erosion; b, The same area in 2007, showing a significant reforestation (alder: *Alnus cremastogyne Burk*) impact. c-e, Reforestation of slash pine (*Pinus elliottii)* on the slope agriculture fields in 2001, 2004 and 2010, respectively.
(TIFF)

S2 Fig Spatial patterns of EVI trends for each grid cell for different season over the period 2000–2009 in southern China. (TIF)

S3 Fig Land cover change on the areas that EVI increased and decreased significantly from 2000 to 2009. (TIF)
