# Supplementary figures and images for: Assessing Vegetation Cover Dynamics Induced by Policy-Driven Ecological Restoration and Implication to Soil Erosion in Southern China
Source: PLoS One. 2015 Jun 26;10(6):e0131352. doi: 10.1371/journal.pone.0131352 (PMC4482633; doi:10.1371/journal.pone.0131352)

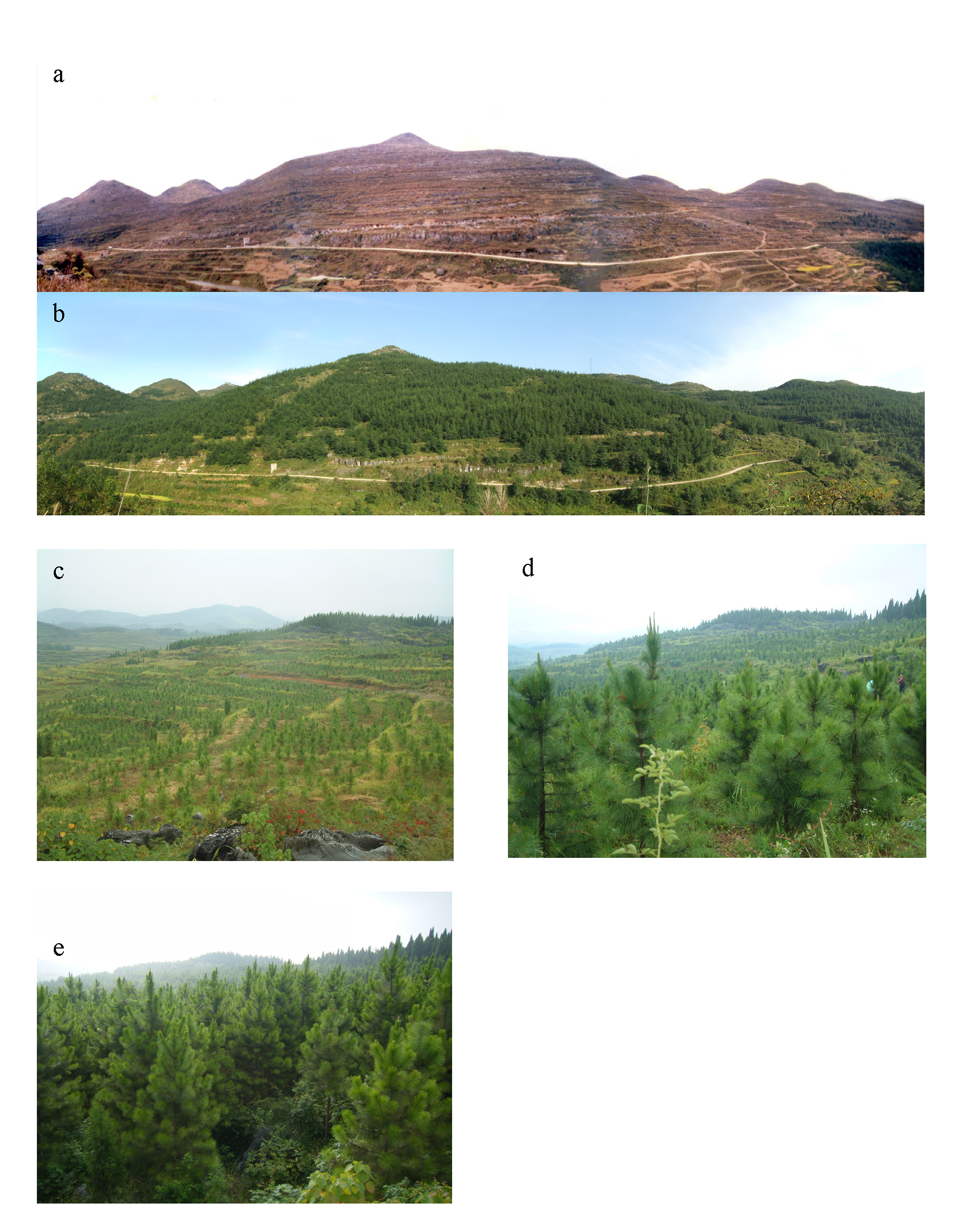

Supplement: S1 Fig — a, Less productive agricultural fields on the slopes before 2001 with severe soil erosion; b, The same area in 2007, showing a significant reforestation (alder: Alnus cremastogyne Burk) impact. c-e, Reforestation of slash pine (Pinus elliottii) on the slope agriculture fields in 2001, 2004 and 2010, respectively. (TIFF) [file pone.0131352.s001.tiff]

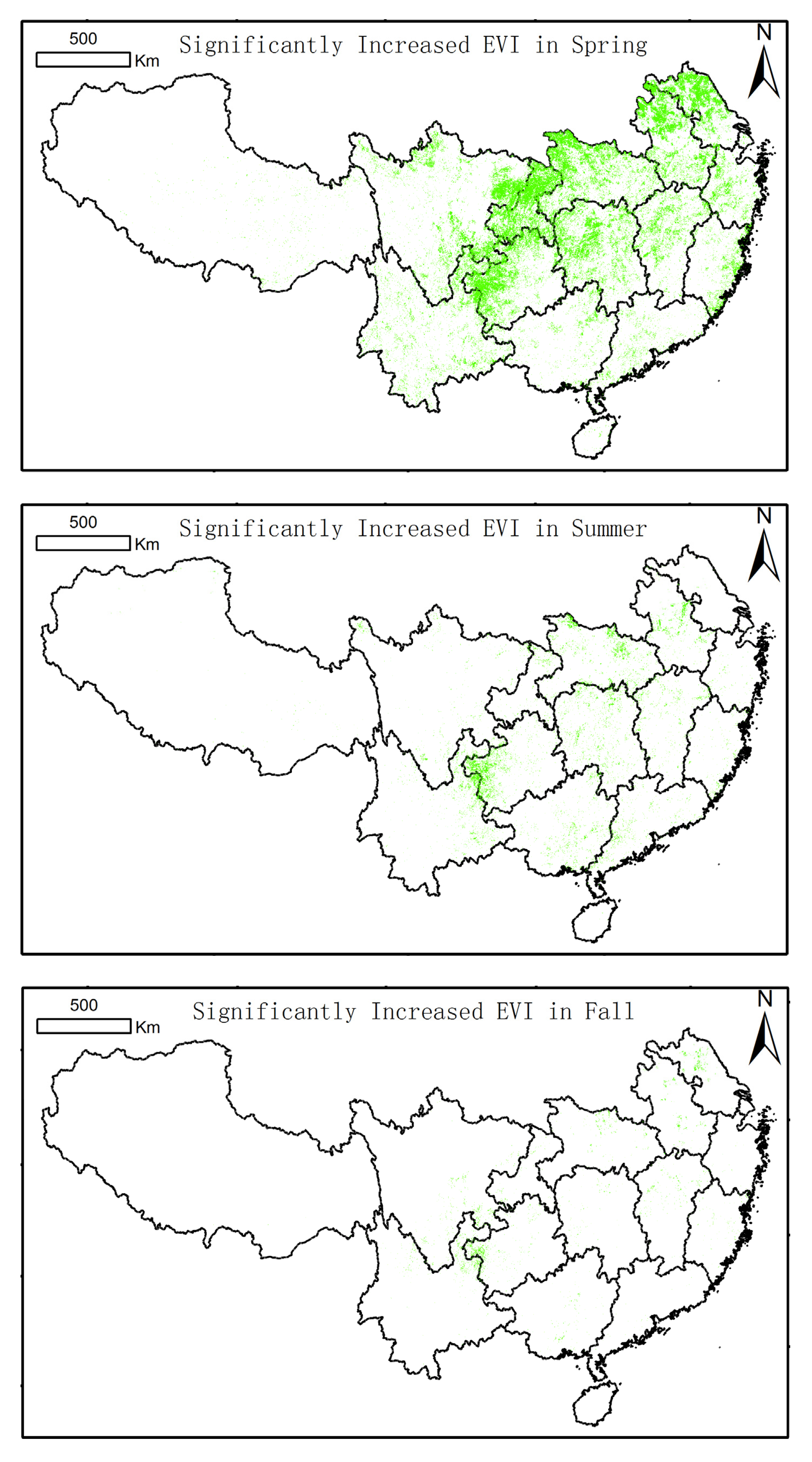

Supplement: S2 Fig — (TIF) [file pone.0131352.s002.tif]
